# Claudin-7与Slug在肺鳞癌和腺癌中的表达及其临床意义

**DOI:** 10.3779/j.issn.1009-3419.2011.06.03

**Published:** 2011-06-20

**Authors:** 锐 李, 道荣 张, 存伟 蔡, 经宇 董

**Affiliations:** 1 110001 沈阳，中国医科大学基础医学院病理教研室 Department of Pathology, China Medical University, Shenyang 110001, China; 2 110042 沈阳，辽宁省肿瘤医院病理科 Department of Pathology, Liaoning Cancer Hospital, Shenyang 110042, China

**Keywords:** 肺肿瘤, Claudin, Slug, 紧密连接, Lung neoplasms, Claudin, Slug, Tight junction

## Abstract

**背景与目的:**

Claudins是紧密连接的骨架蛋白，Claudin-7是Claudins家族成员之一。本研究旨在观察Claudin-7和Slug在肺鳞癌和腺癌中的表达及其与临床病理因素的关系，并探讨Claudin-7和Slug的相互关系。

**方法:**

采用免疫组织化学SP法检测101例原发性肺鳞癌、腺癌组织中Claudin-7和Slug的表达，同时应用Western blot检测30例新鲜肺癌组织及其配对的癌旁组织中Claudin-7和Slug的表达情况。

**结果:**

Claudin-7在肺癌中的表达明显低于正常肺组织，并且与分化程度和淋巴结转移有关（*P* < 0.05），Slug在肺癌中的表达明显高于正常肺组织，除与分化程度和淋巴结转移有关外，还与TNM分期有关（*P* < 0.05），肺鳞癌、腺癌中Claudin-7与Slug的表达具有负相关性（*r*=-0.566, 8）。

**结论:**

肺鳞癌、腺癌中Claudin-7的低表达与Slug的高表达可能是肺组织恶性转变和转移的有关标志物之一。

## Abstract (en)

**Background and objective:**

The aim of this study is to investigate the expression of Claudin-7, Slug and their correltion with clinicopathological characteristics in lung squamous cell carcinoma and adenocarcinoma. We also investigated the relationship between the two factors.

**Methods:**

The expressions of Claudin-7 and Slug in proteins were detected in 101 cases of lung squamous cell carcinoma and adenocarcinoma samples by immunohistochemistry SP method, and Western blot was applied to detect the expressions of Claudin-7 and Slug in 30 specimens of fresh lung cancer and corresponding paracancerous tissues.

**Results:**

The expression of Claudin-7 was remarkably decreased in squamous cell carcinoma and adenocarcinoma tissues compared with normal lung tissue, and its expression level was closely correlated with differentiation grade and lymphatic metastasis (*P* < 0.05), whereas the expression of Slug was significantly higher in cancer tissues than that in normal lung tissue. Outside differentiation grade and lymphatic metastasis, the expression of Slug was related to TNM stage (*P* < 0.05). The expression of Claudin-7 was negatively correlated with the expression of Slug in lung squamous cell carcinoma and adenocarcinoma (*r*=-0.566, 8).

**Conclusion:**

The down-regulation of Claudin-7 and overexpression of Slug might be one of pertinent biological markers for malignant transformation and metastasis of lung squamous cell carcinoma and adenocarcinoma.

Claudins是紧密连接的骨架蛋白，参与维持紧密连接的选择渗透性和细胞的极性，至今发现有24个亚型，主要分布于皮肤、脑、神经系统及内脏组织中。Claudin-7是Claudins家族成员之一，调节肾细胞间侧Cl^-^和Na^+^的渗透性。起初Claudin-7是作为抑癌基因被发现的，但目前的研究发现其在肿瘤中的表达存有差异性，如食管癌^[1]^中Claudin-7的表达降低或缺失可促进肿瘤的侵袭和转移，但在胃腺癌^[2]^和卵巢癌^[3]^中Claudin-7的表达较周围正常组织升高，并且升高的Claudin-7与肿瘤的浸润深度和局部淋巴结转移呈正相关。

锌指转录因子Snail和Slug是上皮-间质转化（epitheli-al-mesenchymal transition, EMT）的重要调节因子，通过与靶基因启动子上的E-box结合抑制蛋白转录，诱导EMT，使肿瘤细胞变得具有迁移性和侵袭性。在食管鳞癌和乳腺癌细胞系中Snail及其同系物SNAI1P可通过与Claudin-7启动子上的E-box结合抑制其转录^[4, 5]^。Manzanares等^[6]^发现，Slug与Snail具有高度的同源性，在N末端都含有调控Claudin-7的关键序列SNAG反式转录区，并且在人类Clau-din-7启动子上含有7个E-box，为Slug的转录抑制提供了结合位点，那么Slug是否能调控Claudin-7的表达呢？

本实验拟采用免疫组织化学SP方法和Western blot检测Slug与Claudin-7蛋白在肺癌组织、癌旁正常肺组织的表达情况，分析不同类型肺癌组织中Slug与Claudin-7的表达差异性，以及与其临床病理因素之间的关系，并初步探讨这两种蛋白表达的相关性。

## 1 材料与方法

### 1.1 标本

101例原发性肺癌组织和40例癌旁肺组织均来自中国医科大学附属第一医院2004年8月-2010年5月外科手术切除标本，其中男性60例，女性41例；平均年龄56.8岁；所有患者术前均未接受放、化疗。标本经10%中性甲醛固定，石蜡包埋，HE常规染色后明确诊断。分别由两名病理医师根据2003年WTO肺癌分类标准和TNM分期系统，确定肿瘤的组织类型和临床病理分期。包括：鳞癌52例，腺癌49例；高分化33例，中分化42例，低分化26例；有淋巴结转移者63例，无淋巴结转移者38例；Ⅰa期15例，Ⅰb期19例，Ⅱa期21例，Ⅱb期18例，Ⅲa期13例，Ⅲb期9例，Ⅳ期6例。

30对新鲜肺癌组织及其癌旁肺组织（远离肿瘤至少5 cm）取自中国医科大学附属第一医院2009年6月-2010年5月手术切除标本。术中切除后立即置于-70 ℃冰箱保存，用于提取组织蛋白。

### 1.2 主要试剂

浓缩型兔抗人Claudin-7多克隆抗体（34-9100）购自美国Invitrogen公司，兔抗人Slug多克隆抗体（bs-1382R）购自北京博奥森生物技术有限公司。SP免疫组织化学试剂盒购自福州迈新生物技术开发公司。

### 1.3 实验方法

#### 1.3.1 免疫组织化学

组织标本经10%中性甲醛溶液固定，石蜡包埋，制成4 μm切片。采用免疫组化SP法检测肺鳞癌、腺癌组织中Claudin-7（1:200）和Slug（1:150）蛋白表达。以PBS代替一抗作阴性对照，用已知阳性肺癌切片作阳性对照。

#### 1.3.2 Western Blot

取50 mg左右大小的组织块（包括肺癌组织及配对癌旁肺组织），放入500 μL裂解液中冰浴下匀浆，4 ℃静置20 min，低温高速离心（4 ℃，15, 000转/分，15 min），收集上清。加样，上样总蛋白量80 μg。10%SDS电泳，浓缩胶电压80 V，分离胶电压120 V。将蛋白转印到PVDF膜上（50 V，4 ℃湿转120 min）。5%脱脂奶粉封闭2 h，加兔抗人多克隆Claudin-7抗体（1:300）和兔抗人多克隆Slug抗体（1:300），4 ℃孵育过夜，TTBS漂洗3次后，加二抗温育2 h，TTBS漂洗3次后ECL发光。结果经自动凝胶成像分析仪采集，进行灰度值测定。实验至少重复3次，取平均值。

### 1.4 结果判定

#### 1.4.1 免疫组织化学结果判定

细胞内呈现棕黄色细小颗粒者为阳性染色，每张切片在400倍镜下观察10个视野，每个视野计数100个肿瘤细胞。阳性细胞数≤10%为1分，11%-50%为2分，50%-75%为3分，≥75%为4分；染色强度评分：无色为0分，淡色为1分，棕黄色为2分，棕褐色为3分；最后按照乘积分数分为4个等级：“-”（0-2），“+”（3-4），“++”（6-8），“+++”（9-12）。结果判定：分数≥3定为阳性， < 3为阴性。

#### 1.4.2 Western blot结果判定

Claudin-7条带在22 kDa处，Slug条带在30 kDa处，利用Image J软件半定量分析特异性条带的光密度值，取其与β-actin的比值作为相对表达量进行比较分析。

### 1.5 统计学分析

采用SPSS 13.0统计学分析软件，对Clau-din-7和Slug表达与临床病理因素的关系用χ^2^检验；Claudin-7和Slug表达的关系采用*Spearman*等级相关分析，采用*t*检验分析Western blot图像灰度值。*P* < 0.05为有统计学差异。

## 2 结果

### 2.1 免疫组织化学结果

#### 2.1.1 Claudin-7与Slug在正常肺组织和肺癌组织中的表达

Claudin-7在40例正常肺组织中高表达，表达率为95%（38/40），表达主要集中在细胞膜和细胞浆；Slug在正常肺组织中低表达，表达率为37.5%（15/40），以胞浆表达为主（图 1）。在101例肺癌组织中Claudin-7阳性表达58例（57.4%），表达主要定位于细胞浆，少量定位于细胞核，在肺鳞癌和腺癌中的表达较正常肺组织明显减弱；Slug阳性表达83例（75.8%），表达主要定位于细胞浆和细胞核，在肺鳞癌和腺癌中的表达较正常肺组织明显增高（图 1）。随着分化程度的降低，Claudin-7的表达逐渐减弱甚至缺失，而Slug表达则逐渐增强，并在低分化的癌组织中出现较强的核表达。

**1:**
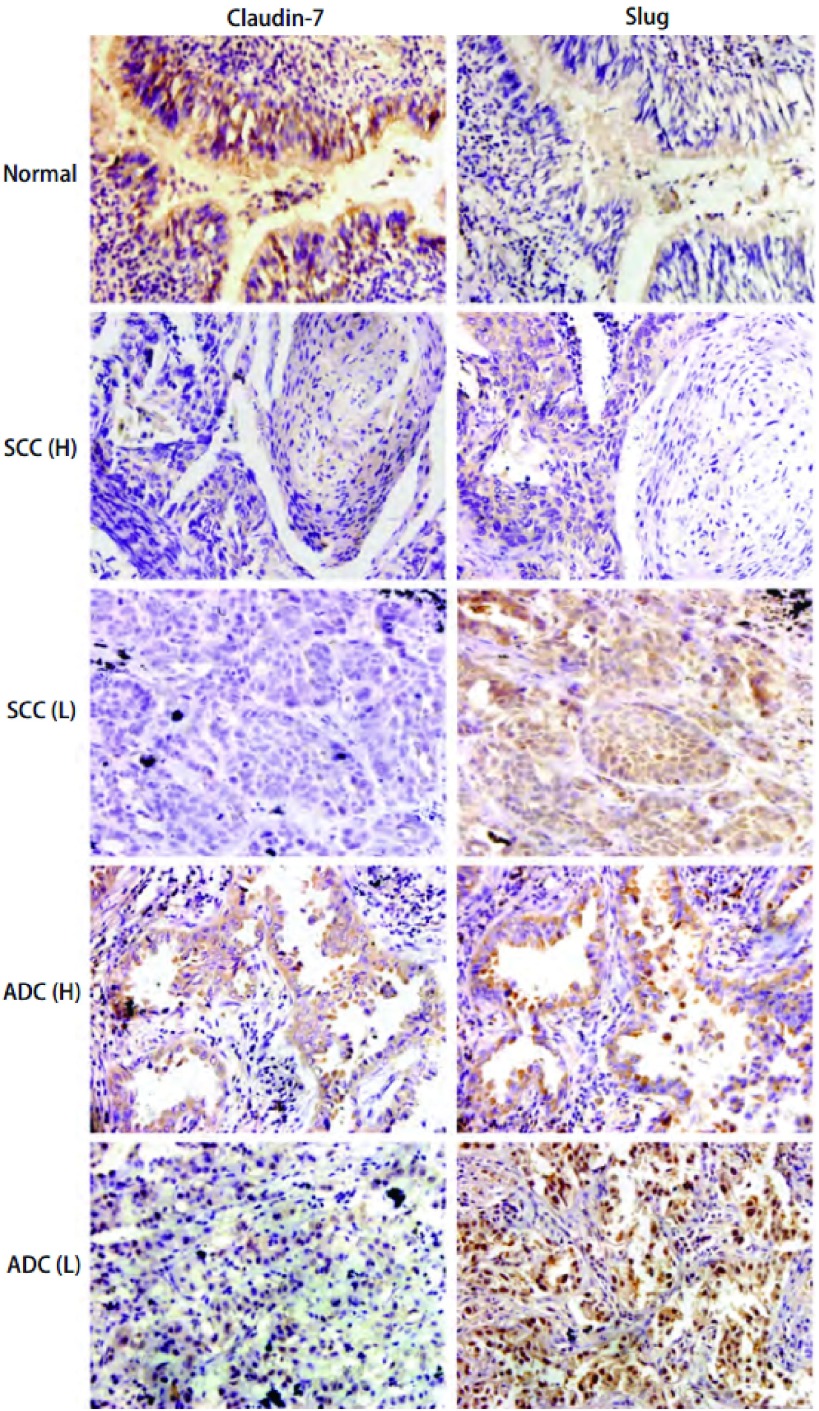
Claudin-7与Slug在肺癌组织中的表达（SP，×400）。SCC：鳞癌；ADC：腺癌；H：分化较好；L：分化较差。 The expression of Claudin-7 and Slug in lung cancers (SP, ×400). SCC: lung squa-mous cell carcinoma; ADC: adenocarcinoma; H: well differentiated; L: poorly differentiated.

#### 2.1.2 Claudin-7和Slug蛋白表达与肺癌临床病理因素的关系

Claudin-7与Slug蛋白在肺鳞癌和腺癌中的表达情况详见表 1。101例肺鳞癌和腺癌中Claudin-7的阳性表达率与组织分化程度及淋巴结转移有密切关系。而Slug除与组织分化程度和淋巴结转移有密切关系外，还与TNM分期相关。随着分化程度的降低，Claudin-7的阳性表达率则明显下降（*P*=0.001），有淋巴结转移的肺癌组织中Claudin-7阳性表达率较无淋巴结转移的肺癌组织明显降低（*P* < 0.001）。而随着分化程度的降低Slug的阳性表达率逐渐升高（*P* < 0.05），有淋巴结转移的肺癌组织中其阳性表达率较无淋巴结转移的肺癌组织明显增高（*P* < 0.05）。不同TNM分期中Slug的阳性表达率随着分期的升高而升高（*P* < 0.05）。Claudin-7与Slug蛋白的表达与性别、年龄、病理组织学分型均无明显关系。

**1:** Claudin-7和Slug的表达及与临床病理学参数的关系 Associations between expression of Claudin-7 and Slug and clinicopathologic parameters

Characteristic	*n*	Claudirv-7				Slug		
		+	*X*^2^	*p*		+	*X*^2^	*P*
Sex			2.110	0.146			0.479	0.489
Male	60	38				45		
Female	41	20				38		
Age (year)			0.240	0.652			0.905	0.341
> 55	63	35				50		
< 55	38	23				33		
Histology			0.154	0.694			0.813	0.367
SCC	52	32				41		
ACD	49	26				42		
Differentiation			13.39	0.001			8.797	0.012
Well	33	23				22		
Moderate	42	28				39		
Poor	26	7				22		
Lymph node metastasis			17.877	< 0.001			11.172	0.001
N0	38	32				25		
N1-3	63	26				58		
P-TNM status			2.663	0.264			10.792	0.003
Ⅰ	34	23				25		
Ⅱ	39	19				32		
Ⅲ+Ⅳ	28	16				26		

#### 2.1.3 Claudin-7与Slug蛋白表达的相关性

如表 2所示，101例肺癌中42例Claudin-7与Slug同时阳性表达，2例同时阴性表达，Claudin-7阳性表达而Slug阴性表达为16例，Slug阳性表达而Clau-din-7阴性表达为41例，经统计学分析显示Slug与Claudin-7的蛋白表达具有明显负相关性（*r*=-0.566, 8）。

**2:** Claudin-7与Slug在肺癌中表达的关系 Correlation between Claudin-7 and Slug expression

		Slug		*r*	*P*
		+	-		
Claudin-7	+	42	16	-0.5668	< 0.001
	-	41	2		

### 2.2 Western Blot中Claudin-7与Slug在肺癌组织中的表达

Claudin-7和Slug在30对肺癌组织及其癌旁肺组织中均有不同程度的表达（图 2）。其中Claudin-7在癌组织中的表达明显低于癌旁肺组织，平均灰度值分别是1.04±0.23和6.02±1.34（*P*=0.004）。Slug在癌组织中的表达明显高于对应癌旁肺组织，平均灰度值分别是5.83±1.25和2.81±0.48（*P*=0.037）。

**2:**
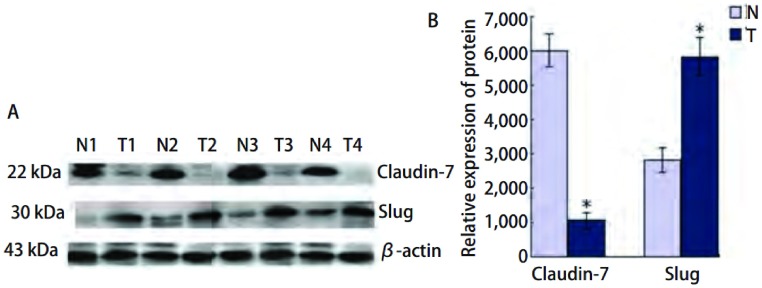
Western blot检测肺癌组织及其癌旁组织中Claudin-7和Slug的表达。A：肺鳞癌、腺癌中Claudin-7和Slug的蛋白表达情况；T1, 2：SCC；T3, 4：ADC；N：癌旁组织；B：Claudin-7和Slug蛋白的相对表达量。^*^：*P* < 0.05。 Expression of Claudin-7 and Slug protein in lung can-cer tissues and paracancerous specimens by Western blot. A: Result of Western blot for expressions of Claudin-7 and Slug in lung quamous cell carcinoma and adenocarcinoma; T1, 2: SCC; T3, 4: ADC; N: paracancerous specimens; B: The histogram of relative expression rate of Claudin-7 protein compared to Slug. ^*^: *P* < 0.05.

## 3 讨论

癌细胞的侵袭转移，首先必须从原发癌灶中脱离出来，而细胞之间粘附结构功能的改变则使恶性肿瘤细胞易于脱离原发组织。Claudins的潜在作用在于限制癌细胞的营养和生长因子供应，在癌中失去紧密连接蛋白在膜上的定位是肿瘤发展过程中的关键步骤。Kominsky等^[7]^发现在乳腺癌组织中Claudin-7的蛋白和mRNA水平降低，并与癌组织级别呈负相关；并且在口腔鳞癌中Claudin-7的表达减少与临床级别和术后复发有关^[8]^。本实验发现Claudin-7在肺鳞癌、腺癌组织中的表达较正常肺组织减弱，并在分化较差和有转移的癌组织中下降较明显，甚至表达缺失。但是在其他肿瘤如胃腺癌^[2]^，卵巢癌^[3]^中，Claudin-7的表达反而升高，并且与肿瘤恶性程度增高有关，这提示Claudin-7在肿瘤中的表达具有组织特异性。本实验还发现Claudin-7在癌组织中以胞浆表达为主，可能与Claudin-7参与细胞分化及其他因子的调控有关^[9, 10]^。奇怪的是Claudin-7在正常肺组织中也出现了较强的胞浆表达，并且在胆囊^[11]^和食管^[10]^上皮基底层中也有类似发现，可能与Claudin-7的表达受周围微环境的影响有关，但具体机制不详。

在EMT中上皮细胞向间质细胞转化，使细胞获得了更强的活动和侵袭能力，从而穿越细胞外基质在血管和淋巴管中停留，启动肿瘤转移的第一步。本研究中，Slug在肺癌中的表达明显增高，随着肺癌细胞分化程度的下降而呈上升趋势，并且与淋巴结转移及TNM分期呈正相关；在结肠癌中Slug的高表达与Dukes分期和远处转移有关^[12]^，这与我们的研究结果一致。但Shih等^[13]^通过RT-PCR在肺腺癌中研究发现，Slug的mRNA高表达与患者的术后复发和较短生存期有关，但是与淋巴结转移无关，可能与其样本量较少有关。

目前关于Claudin-7的调节因子是个研究热点，除Snail与SNAI1P之外，ELF3是Claudin-7正性转录因子，可上调Claudin-7的表达^[14]^，而在正常的结直肠上皮中TCF4通过与SOX-9作用抑制Claudin-7的表达，但是这种抑制作用在肿瘤中却失去了功效^[15]^。在哺乳动物中Slug与Snail具有高度的同源性，两者都通过与启动子上的E-box结合抑制occludin、ZO-1、Claudin-1和E-cadherin的表达进而诱导EMT的发生，说明作为EMT的诱导因子Slug可通过抑制紧密连接蛋白转录促进EMT发生，在本实验中我们发现在分化较差的肺癌组织中Slug主要在核表达，而Claudin-7表达明显减弱甚至缺失，而且统计分析也证实了Slug与Claudin-7在肺癌中的表达具有明显的负相关性（*P* < 0.05），因此我们大胆推测Slug可能通过与Claudin-7启动子上的E-box结合进而调控Claudin-7的表达，但这有待进一步的验证。
